# Supplementary material for: pH-Sensitive Folic Acid Conjugated Alginate Nanoparticle for Induction of Cancer-Specific Fluorescence Imaging
Source: Pharmaceutics. 2020 Jun 11;12(6):537. doi: 10.3390/pharmaceutics12060537 (PMC7355973; doi:10.3390/pharmaceutics12060537)
Supplement: Supplementary file 1 [file pharmaceutics-12-00537-s001.pdf]

pharmaceutics

MDPI

Supplementary Materials: pH-Sensitive Folic Acid Conjugated Alginate Nanoparticle for Induction of Cancer-Specific Fluorescence Imaging

Sara Lee and Kangwon Lee

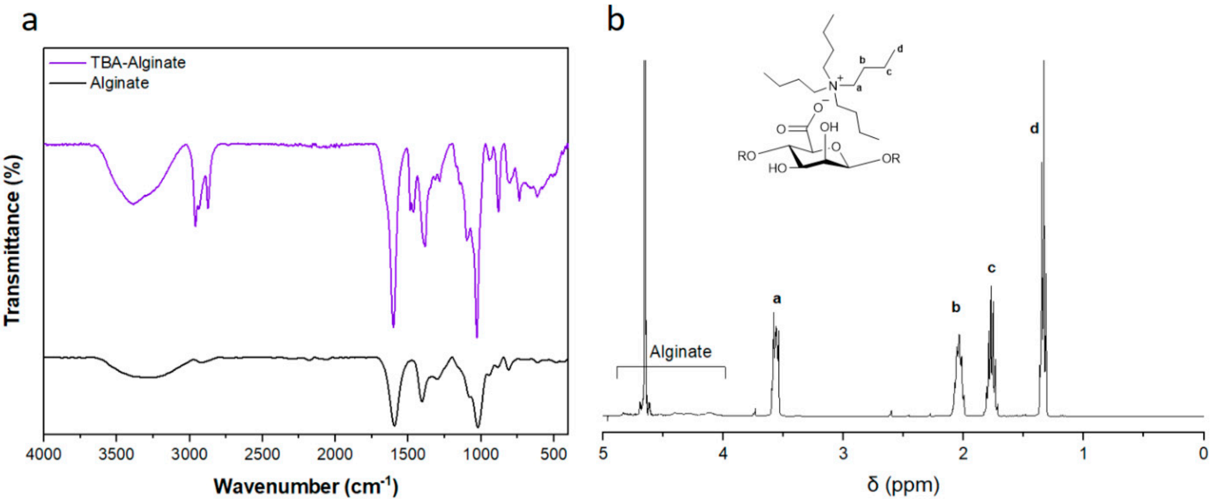

Figure S1. Characterization of TBA-Alginate. (**a**) FT-IR spectra of TBA-Alginate and alginate, (**b**) H^1 NMR spectrum of alginate-TBA in D_2O.

Table S1. Classification by nanoparticle fabrication conditions. The average size, ζ potential, 5-ALA loading capacity (LC) and encapsulation efficiency (EE) of AF nanoparticles.

NP	Alginate-FA (wt%)	HLB	Day 0		Zeta potential (mV)	LC%	EE%
			Size (nm)	PDI			
NP1	0.5	7	117.9±0.65	0.389	-27.4±2.1	1.2%	6.33%
NP2		8	53.56±1.52	0.496	-23.3±0.7	0.4%	8.8%
NP3	1	7	83.45±3.47	0.584	-22.8±2	2.8%	27.14%
NP4		8	45.89±1.56	0.454	-29.3±0.1	1.8%	31.6%

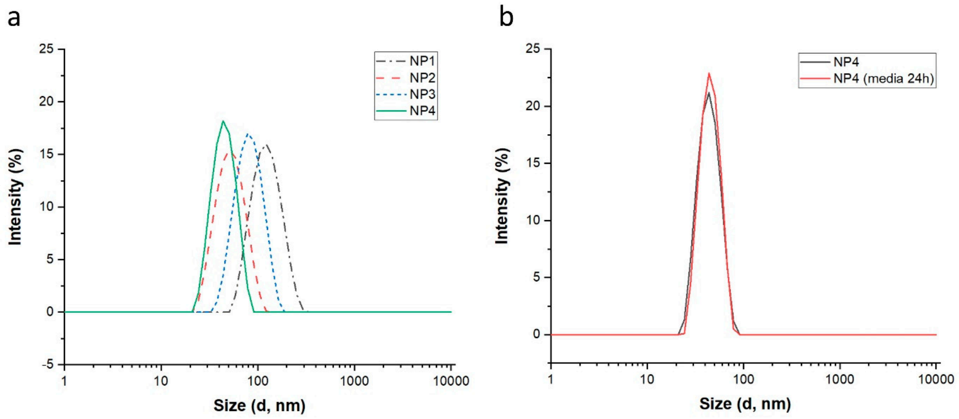

Figure S2. (**a**) The intensity according to the size of the four types of AF NPs was measured and indicated using DLS. (**b**) The size distribution of AF NPs stored in PBS at 4 °C and AF NPs incubated at 37 °C for 24 hours in cell media was shown.

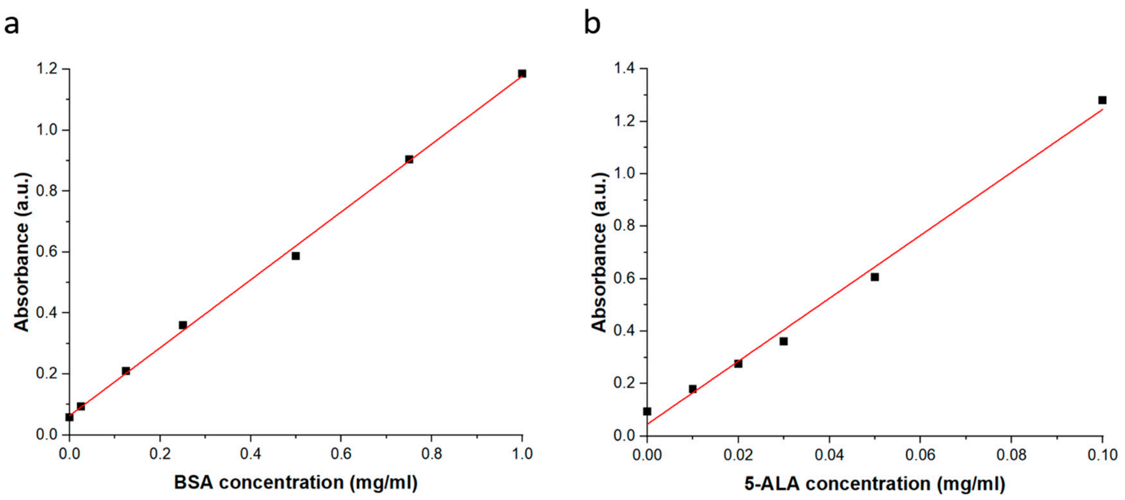

Figure S3. (**a**) The standard curve of BSA for BCA assay. (**b**) The calibration curve of 5-ALA by TBNSA Kit.

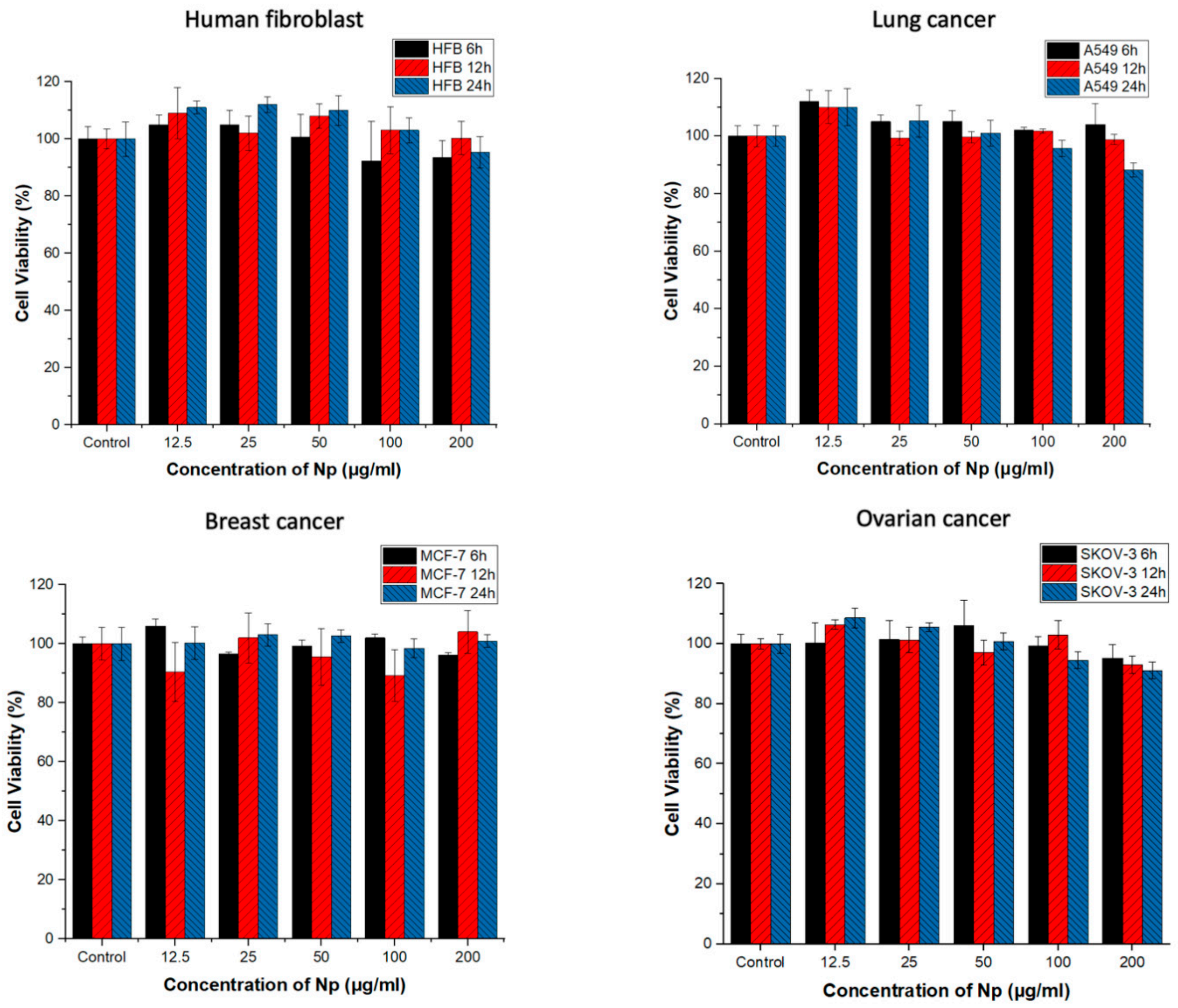

Figure S4. Cytotoxicity test of AF nanoparticles. After treating AF nanoparticles of various concentrations for 6 h, 12 h, and 24 h, the toxicity of the AF nanoparticles was investigated using CCK-8 assay.
